# Sensing, signaling and surviving mitochondrial stress

**DOI:** 10.1007/s00018-021-03887-7

**Published:** 2021-07-06

**Authors:** Eva-Maria Eckl, Olga Ziegemann, Luisa Krumwiede, Evelyn Fessler, Lucas T. Jae

**Affiliations:** Gene Center and Department of Biochemistry, Ludwig-Maximilians-Universität München, Feodor-Lynen-Strasse 25, 81377 Munich, Germany

**Keywords:** Mitochondrial unfolded protein response (UPRmt), Integrated stress response (ISR), Mitochondria, Protein import, Mitophagy, DELE1

## Abstract

Mitochondrial fidelity is a key determinant of longevity and was found to be perturbed in a multitude of disease contexts ranging from neurodegeneration to heart failure. Tight homeostatic control of the mitochondrial proteome is a crucial aspect of mitochondrial function, which is severely complicated by the evolutionary origin and resulting peculiarities of the organelle. This is, on one hand, reflected by a range of basal quality control factors such as mitochondria-resident chaperones and proteases, that assist in import and folding of precursors as well as removal of aggregated proteins. On the other hand, stress causes the activation of several additional mechanisms that counteract any damage that may threaten mitochondrial function. Countermeasures depend on the location and intensity of the stress and on a range of factors that are equipped to sense and signal the nature of the encountered perturbation. Defective mitochondrial import activates mechanisms that combat the accumulation of precursors in the cytosol and the import pore. To resolve proteotoxic stress in the organelle interior, mitochondria depend on nuclear transcriptional programs, such as the mitochondrial unfolded protein response and the integrated stress response. If organelle damage is too severe, mitochondria signal for their own destruction in a process termed mitophagy, thereby preventing further harm to the mitochondrial network and allowing the cell to salvage their biological building blocks. Here, we provide an overview of how different types and intensities of stress activate distinct pathways aimed at preserving mitochondrial fidelity.

## Introduction

Proteotoxicity is a central feature of many age-related diseases, including neurodegeneration and heart disease [1, 2]. The eukaryotic cell defies this dangerous source of stress with multiple layers of protein quality control mechanisms, including tuning of protein production, folding and removal. These can lower the burden on the cell by reducing the rate of translation, stabilizing and folding protein precursors with the help of chaperones, degrading misfolded or aggregated proteins through proteases, and even removal of cellular macro-structures by autophagy [3].

While coordination of these measures is far from simple in the cytosol, enclosed and relatively isolated cellular structures such as the endoplasmic reticulum and mitochondria face additional challenges and require compartmentalization of the cellular quality control processes that combat proteotoxicity [4]. Especially mitochondria possess unique properties that further complicate protein quality control. As relatives of α-proteobacteria [5], mitochondria remained partially autonomous after their engulfment by the progenitor of the eukaryotic cell, which is reflected by their double membrane and their own genome (mtDNA). However, as the mtDNA only encodes a handful of proteins, tRNAs and rRNAs [6], the organelle heavily relies on nuclear genes to fulfill its versatile functions. As a consequence, the majority of mitochondrial proteins—being estimated to encompass more than 1000 proteins in yeast and more than 1500 in humans—need to be transcribed and translated by cytosolic ribosomes [7] prior to their delicate import across one or two of the mitochondrial membranes, depending on the submitochondrial destination [8, 9]. This process needs to run smoothly to assure proper synchronization of the nuclear and mitochondrial genome, which is, for instance, crucial for the correct stoichiometric configuration of multi-protein complexes of the respiratory chain [6, 10]. Given their origin, mitochondria likely used to contain intrinsic stress response mechanisms [11]. This is still apparent from similarities between certain bacterial and mitochondrial heat shock proteins today [12]. However, as with the majority of processes, in the course of evolution mitochondria have also outsourced these important functions to the host cell and no longer encode stress response genes in humans [3]. This precipitated a series of specialized cellular responses to mitochondrial insults that operate at different levels of mitochondrial protein homeostasis (proteostasis).

When protein import into mitochondria becomes overwhelmed, precursors accumulate in the cytosol, a stress termed mPOS (mitochondrial precursor overaccumulation stress) [13]. In turn, the compromised protein import response (mitoCPR) is activated, which promotes mitochondrial extraction and subsequent degradation of proteins stalled in the import pores [14]. To accelerate recovery, the unfolded protein response activated by the mistargeting of proteins (UPRam) increases proteasome activity and reduces overall protein translation [15].

Other types of mitochondrial stress cannot be resolved at the surface of the organelle and require more substantial mito-nuclear coordination. In response to reduced respiration or loss of quality control factors in the matrix, a complex cytoprotective transcriptional program is activated, that was termed the mitochondrial unfolded protein response (UPRmt) in analogy to the unfolded protein response in the endoplasmic reticulum (UPR^ER^) [16]. In nematodes, certain types of mitochondrial dysfunction, including mitochondrial translation perturbations, impairment of oxidative phosphorylation (OXPHOS) and protein misfolding [17], trigger the nuclear accumulation of the activating transcription factor associated with stress (ATFS-1), which is normally degraded in the mitochondrial matrix. In the nucleus, ATFS-1 initiates the UPRmt, which—among other adaptations—entails upregulation of mitochondrial chaperones and proteases to resolve the stress [18–20]. Interestingly, it was observed that the predominant response to similar types of mitochondrial stress in mammals relies on a different program, known as the integrated stress response (ISR) [21–23]. The ISR is induced by a variety of environmental stimuli that activate one of four known eIF2α kinases—HRI, PKR, PERK and GCN2—and results in a transient attenuation of translation and the induction of a nuanced transcriptional response that can facilitate stress recovery or doom cells to undergo programmed cell death [24].

Mitochondrial stresses that irreversibly perturb mitochondrial function require removal of the damaged part of the organelle to avoid the accumulation and spread of defects and to protect the remainder of the mitochondrial network. While the initial steps of this cascade are once again controlled by mitochondria-localized factors and pathways, ultimately the organelle is destined for engulfment by the autophagosome machinery and its breakdown in the lysosome [25].

The multi-layered nature of mitochondrial stress response pathways underlines the importance of an appropriate response to different degrees and types of stress that can be experienced by this organelle. Interdependence and crosstalk between these mechanisms are only gradually being recognized [3, 26]. Their deregulation or faulty execution can result in long-term mitochondrial dysfunction and ultimately cell death [4]. Apoptosis can also be deliberately induced by certain mitochondrial stress response mechanisms in an attempt to avert further damage to the tissue or organism. At the same time, such a loss of cells can be problematic in tissues with a low regenerative capacity, such as the adult nervous system [27], and likely contributes to disease pathology in various neurodegenerative disorders [28, 29]. Particularly during aging, proteostasis declines due to increased proteotoxic stress, reduced quality control factors and stress response signaling, or a combination of both [1]. The resulting mitochondrial dysfunction is especially problematic in cells like cardiomyocytes and neurons, as those have an increased energy demand to fulfill their functions, rationalizing the increased incidence of diseases arising from these tissues with older age [30, 31]. Conversely, reduced mitochondrial activity during development can benefit longevity [32]. Thus, understanding how mitochondrial defects are sensed, signaled and resolved will be imperative for the design of future strategies to combat aging and age-related afflictions.

In this review, we illuminate a variety of mechanisms that ensure mitochondrial homeostasis in response to distress of varying nature and intensity: from local factors that act on the mitochondrial import pore, to nuclear-encoded pathways that respond to mitochondrial dysfunction such as the UPRmt and the ISR and finally mitophagy, the cellular program that can eliminate faulty mitochondria when the stress is too severe.

## Surveillance of mitochondrial protein import

### Mitochondrial protein import pathways

In contrast to the endoplasmic reticulum (ER), where protein import occurs across a single lipid bilayer [33], the mitochondrial import process needs to differentiate between multiple distinct final destinations within the organelle: the outer (OMM) and inner mitochondrial membrane (IMM), the intermembrane space (IMS), or the matrix. Mitochondria also harbor large multi-protein complexes, whose translation is jointly accomplished by both cytosolic and mitochondrial ribosomes [34]. While it was long thought that the ER represents the sole cellular compartment that can form disulfide bonds, we now know that these structural features can also be produced in the IMS by an unrelated system [35, 36]. These and other challenges are met by an elaborate protein import machinery that encompasses multiple import pathways orchestrating the subcompartment localization and appropriate processing of mitochondrial precursors [8, 9].

Mitochondrial protein import is an ancient, conserved process. The majority of mitochondrial proteins that are translated in the cytosol are bound by heat shock protein 70 and 90 family members, aided by co-chaperones and accessory components, to keep them in an unfolded, import-competent conformation and escort them to the translocase of the outer membrane (TOM) [37–40]. To initiate translocation across the OMM, the TOM complex has three receptor proteins for the recognition of different mitochondrial precursors: Tom20, Tom22 and Tom70. Tom20 and Tom22 are closely associated with Tom40 β-barrel proteins, which represent the channel forming units of the TOM complex that translocates precursors across the OMM [41]. Tom70 more loosely associates with the TOM complex. It was shown to recognize hydrophobic precursors with non-cleavable presequences like membrane proteins of the OMM and IMM [42]. Additionally, Tom70 is able to interact with Hsp70/Hsp90 chaperones [38] and co-chaperones [43], and has recently been proposed to thereby primarily serve as a safeguard against proteotoxicity of hydrophobic precursors in the cytosol [44].

The import of precursors containing non-cleavable mitochondrial targeting sequences (MTS) is mediated by different sorting mechanisms, informed by the respective nature of the precursor. Hydrophobic OMM β-barrel precursors are threaded through the Tom40 channel led by a β-hairpin targeting signal made up of the most C-terminal β-strands of the protein [45]. Polytopic α-helical carrier proteins of the IMM have multiple targeting signals spread over the length of the protein and are translocated through Tom40 in a loop formation with the N- and C-termini following the rest of the protein [46]. Once they emerge from the TOM complex, both of these types of hydrophobic precursors are bound by small chaperones of the IMS and are either delivered to the sorting and assembly machinery (SAM) for insertion into the OMM (β-barrel proteins) or to the translocase of the inner membrane (TIM) 22 complex, the translocase for metabolite carriers of the IMM [8, 47–50]. α-helical transmembrane proteins of the OMM follow different routes of membrane insertion depending on the protein topology [51]. For signal-anchored and polytopic OMM precursors, the transmembrane helix serves as a targeting signal, which is recognized by Tom70. In yeast, the import does not always require Tom40, as Tom70 can also hand the precursor over to the mitochondrial import (MIM) complex for insertion into the OMM [52, 53]. A functional counterpart for the MIM complex has not yet been described for mammalian cells. Besides these mechanisms, certain OMM proteins require a unique composition of the above-mentioned cofactors and protein complexes for their membrane insertion [54, 55]. In contrast, the majority of tail-anchored OMM proteins are thought to be embedded solely based on the lipid composition of the membrane without further assistance of protein complexes [56, 57]. The import of cysteine-rich IMS proteins does not require any of the above-mentioned TOM receptor proteins. For these proteins, a hydrophobic stretch and a cysteine residue serve as IMS targeting signal [58, 59]. After emerging from the Tom40 channel, the targeting signal is recognized by the IMS import and assembly machinery (MIA) [58]. The MIA system not only functions in the translocation of the entire protein into the IMS but also establishes the disulfide bonds on its substrates [35, 60].

In contrast to the above-summarized precursor types with embedded MTS motifs that remain part of the matured proteins, the majority (~ 60%) of mitochondrial precursors bear a cleavable N-terminal MTS that forms an amphiphilic helix with a positive net charge, which is removed in the process of sorting and maturation [61, 62]. This type of MTS, which is also known as the presequence, is recognized by the receptors Tom20 and Tom22, which hand over the precursors to Tom40 [63–65]. After passing through Tom40 led by the N-terminus, these proteins follow the ‘presequence pathway’ via TIM23. TIM23 not only represents the entry gate to the matrix, but can also mediate lateral sorting of α-helical transmembrane proteins into the IMM [66]. The subunit Tim50 serves as primary acceptor of the incoming precursor and as an adapter to the main import pore formed by the Tim23 protein [67–69]. During import, TOM and TIM23 are thought to be able to form a supercomplex which allows precursors to simultaneously pass through both channels [68, 70]. The inner membrane potential (∆Ψm), which is sustained by the electron transport chain, passively promotes the import across the IMM by electrophoretic attraction of the positively charged MTS [71, 72]. Moreover, the conformation of Tim23 is voltage dependent, further emphasizing the role of ∆Ψm in protein import [73–75].

For sorting into the IMM, an internal hydrophobic sequence of the precursor serves as a stop-transfer signal, stalling the import in the Tim23 channel and facilitating the lateral release into the lipid bilayer [76]. If a laterally released protein also contains a cleavage site for the inner membrane peptidase (IMP), it will ultimately mature into a soluble IMS protein [77]. While, from an energetic perspective, the membrane potential suffices for the import of laterally released proteins [76], proteins without a hydrophobic sorting signal require additional mechanisms to reach the matrix in their entirety [78]. The presequence translocase-associated motor (PAM) awaits such precursors at the matrix-facing opening of the Tim23 pore [79]. The inward driving force is generated by ATP hydrolysis through mtHsp70 [80, 81]. Once precursors emerge into the matrix, the mitochondrial protein peptidase (MPP) cleaves off the MTS, which is subsequently degraded [82–84]. Other proteases and peptidases can remove additional, potentially destabilizing residues from the preprotein [85, 86]. Finally, folding into the mature conformation is supported by mitochondrial chaperones [78, 87].

### Mitochondrial import stress—lessons from yeast

Due to its high complexity, the mitochondrial import process is under steady surveillance by multiple quality control mechanisms of the cell, which can be escalated to more substantial rescue programs in the context of mitochondrial perturbation. One such quality control mechanism acts on translating ribosomes in the cytosol or at the mitochondrion. Aberrant or damaged mRNA can result in stalling of the translating ribosome, leading to unproductive translation complexes and, therefore, to truncated and potentially toxic proteins [88]. To avert harm and rescue such non-functional ribosomes, they are split into the 60S and 40S subunits and the mRNA is degraded prior to breakdown of the nascent polypeptide chain and recycling of the 60S subunit in a process termed ribosomal quality control (RQC) [89]. The nascent polypeptide on the 60S ribosomal subunit is ubiquitinated by the E3 ligase Ltn1 and after release of the conjugated tRNA by Vms1, the polypeptide is targeted for proteasomal degradation by the heterotrimeric Cdc48-Ufd1-Npl4 protein complex [88]. In some cases, lysine residues required for ubiquitination might be buried inside the ribosomal tunnel and thus remain inaccessible to Ltn1. Therefore, a process called CAT-tailing, performed by Rqc2, adds alanine and threonine residues to the C-terminal end of the polypeptide chain, thereby elongating the polypeptide and eventually exposing lysine residues for ubiquitination by Ltn1 [90]. CAT-tails also destabilize the polypeptide chain, facilitating their rapid proteasomal degradation, which is essential as CAT-tailed proteins are themselves prone to aggregation [91, 92]. RQC was shown to take place on cytosolic as well as ER- and mitochondria-associated ribosomes [93, 94]. Besides the above-described classical post-translational protein import into mitochondria, there is also evidence that import of nascent polypeptides can occur in a co-translational manner [95]. Therefore, stalled translation complexes can result in the obstruction of the import pore, which requires RQC to be resolved. However, the CAT-tailing process can become detrimental to mitochondria, as proteins that are inaccessible to the cytosolic degradation machinery during their co-translational import can still translocate into mitochondria and may subsequently aggregate within the matrix in a CAT-tail-dependent manner. It has been reported that in ‘mitoRQC’, Vms1 plays an additional role, by displacing Rqc2 and thereby inhibiting the CAT-tailing process. Nonfunctional peptides lacking CAT-tails are then imported and taken care of by mitochondrial protein degradation mechanisms [93].

**Fig. 1:**
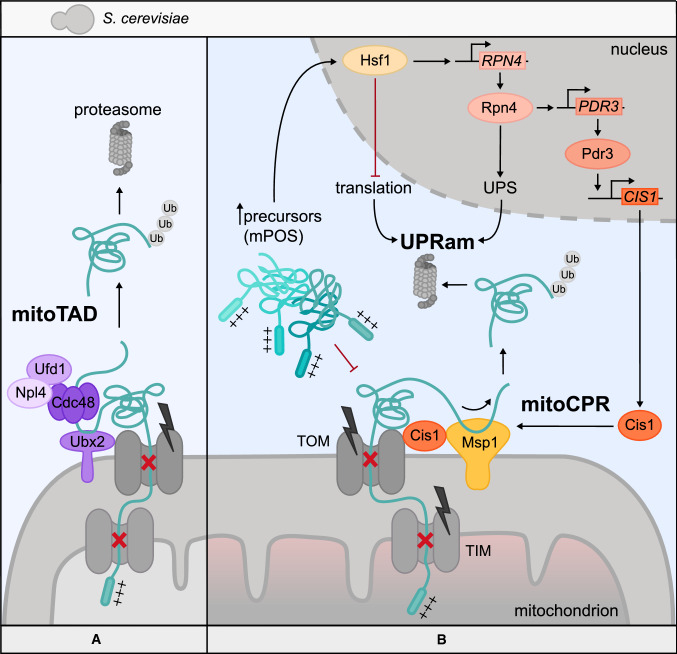
Surveillance of mitochondrial protein import in yeast. Yeast cells developed steady-state quality control mechanisms as well as transcriptional stress responses to safeguard the mitochondrial protein import process and sustain proteostasis. **A** The mitochondrial translocation-associated degradation (mitoTAD) mechanism constitutively monitors the TOM complex under homeostatic conditions. Ubx2 recruits the Cdc48–Ufd1–Npl4 protein complex to the import pore to remove arrested precursors and deliver them to the proteasome for degradation. **B** Severe import defects require further mechanisms to prevent collapse of the import process. The mitochondrial compromised protein import response (mitoCPR), a process active at the site of the translocase, entails the Pdr3-dependent expression of Cis1, an adapter protein that recruits the AAA-ATPase Msp1 to the TOM complex for the extraction of arrested precursors from the translocase and subsequent proteasomal degradation. Grave import defects also result in mitochondrial precursor overaccumulation stress (mPOS) in the cytosol. As a response, yeast cells activate a program called unfolded protein response activated by the mistargeting of proteins (UPRam). The transcription factors Hsf1 and Rpn4 mediate the reduction of cytosolic protein synthesis, expression of heat shock proteins and an increased proteasomal degradation of proteins. MitoCPR and UPRam are interconnected through Rpn4, which promotes UPRam, as well as initiation of mitoCPR to eliminate arrested precursors form the mitochondrial import channel

Another steady-state quality control mechanism that safeguards mitochondrial protein import acts at the level of the import pore: precursors that are in the process of being translocated through the TOM complex are subject to constitutive monitoring and so-called mitochondrial translocation-associated degradation (mitoTAD) [96]. In mitoTAD, a new role for the protein Ubx2, which is known from ER-associated degradation [97], has been identified. A non-ER-associated population of Ubx2 is anchored in the OMM and interacts with Tom70. This interaction is strengthened in the presence of arrested precursors, which are recognized by the UBA domain of Ubx2. The UBX domain, on the other hand, recruits the aforementioned Cdc48-Ufd1-Npl4 protein complex to the TOM channel to promote extraction of stalled precursors from the pore and enable their proteasomal degradation (Fig. 1A). Interestingly, yeast double knockout mutants for Ubx2 and the RQC-protein Vms1 display a severe growth defect, while the single deletion of either gene has little effect on overall fitness. These findings indicate that different steady-state quality control mechanisms at the mitochondrial import pore are functionally linked, yet able to partially compensate for one another [96].

When steady-state quality control mechanisms are rendered insufficient, additional programs are set off to maintain cellular proteostasis. An overabundance of mitochondrial precursors that exceeds the capacity of the mitochondrial translocation machinery can lead to their arrest within the import channel. Yeast cells respond to such arrested precursors by triggering the mitochondrial compromised protein import response (mitoCPR), which was initially discovered through the overexpression of single-pass transmembrane proteins of the IMM (Fig. 1B) [14]. This transcriptional response is coordinated by Pdr3, a transcription factor primarily known as a master mediator of multidrug resistance [98]. Pdr3 has previously also been described to alter gene expression following electron transport chain defects and mtDNA loss [99]. One of the upregulated genes following import inhibition by overexpression of IMM proteins is the peripheral outer membrane protein Cis1, which binds Tom70 and recruits the AAA-ATPase Msp1 to the cytosolic side of the TOM complex. Msp1 was proposed to extract the arrested proteins from the pore for subsequent proteasomal degradation, which is reminiscent of its role in the removal of mistargeted tail-anchored proteins from the OMM [100, 101]. However, whether clearance of stalled precursors is sufficient for the resumption of translocation across the pore has yet to be clarified.

Inhibition or overloading of different key players of the translocation and sorting machinery not only leads to precursor arrest in the translocases, but subsequently also results in mitochondrial precursor overaccumulation stress (mPOS) in the cytosol, a form of protein folding stress (Fig. 1B) [13, 15]. mPOS was initially identified by the overexpression of a misfolding mutant of the IMM ADP/ATP carrier protein Aac2 (Aac2-A128P), which is sorted by TIM22. However, overexpression of the wildtype version of Aac2 similarly generates mPOS by overwhelming TIM22 [13]. This phenomenon is not limited to the IMM carrier translocase TIM22, as overexpression of IMM proteins with an α-helical stop-transfer signal, which are clients of TIM23, also results in cytosolic accumulation of their precursors. Even matrix targeted precursors, which are TIM23 substrates as well, were found to accumulate outside mitochondria [14]. Hence, sorting at the IMM appears to be a major bottleneck of mitochondrial protein import. mPOS was found to induce a cytosolic stress response of elevated proteasome activity and global attenuation of translation, a phenomenon termed UPR activated by mistargeting of proteins (UPRam) [13, 15]. Increased protein degradation activity is due to elevated proteasome assembly following precursor accumulation, which was also observed in yeast conditional mutants of import-related proteins such as Mia40 or Pam16/18 (TIM23 complex). Decrease in overall protein synthesis seems to result from reduced expression of cytosolic ribosome components [15].

These features of mPOS, UPRam and mitoCPR could recently be integrated into a broader transcriptional response with the help of comprehensive RNAseq experiments [102]. According to the model of mitoprotein-induced stress response, the observed translation inhibition and proteasome activation are mediated by the transcription factors Hsf1 and Rpn4: In an early stage after the induction of import stress, the general heat shock response factor Hsf1 activates classical heat shock response genes including the Hsp70, Hsp90 and Hsp40 chaperone families. Mitochondrial precursors rely on these chaperones to keep them in an unfolded conformation required for import [37]. Simultaneously, Hsf1 inhibits the synthesis of cytosolic ribosomal proteins and co-translational chaperones. Another target of Hsf1 is Rpn4, which in turn activates the transcription of proteins of the ubiquitin proteasome system (UPS) [102]. Taken together, Hsf1 and Rpn4 explain the UPRam phenotype at the transcriptional level.

Remarkably, Rpn4 also induces the transcription of Pdr3, the factor that mediates expression of Cis1 (Fig. 1B) [14]. This observation connects the global, cytosolic stress response following precursor overaccumulation with the local stress response at the import pore, supporting the idea that mitoCPR is only induced after hampered protein import has already led to severe consequences in the cytosol which trigger Hsf1. Given the existence of mitoTAD, it seems that the cell has developed different, partially redundant solutions to the same problem, which are adapted to the severity of the import block. This raises the question how these pathways are coordinated when they are both active. Do the two unclogging-complexes compete at the site of the translocon under these circumstances? Alternatively, mitoTAD may become actively replaced by mitoCPR during prolonged import stress.

Interestingly, recent work also revealed a metabolic aspect of defective or overloaded protein import: OXPHOS and tricarboxylic acid cycle (TCA) components can be downregulated in the context of import stress independently of Hsf1 and Rpn4 [102]. The resulting decrease in mitochondrial respiration was found to be mediated by inactivation of the heme activator protein (HAP) complex. This metabolic master regulator of yeast is known for its influence on the TCA cycle, OXPHOS, mitochondrial protein import and mitochondrial protein translation [103, 104]. Thus, the cytosolic and the intramitochondrial responses to import defects seem to operate simultaneously but independently of one another. The former is likely mediated by mPOS, whereas the latter might be activated by the depletion of nuclear-encoded proteins from mitochondria. This leads to mito-nuclear imbalance, reflecting a disproportion of mitochondrial and nuclear-encoded subunits of protein complexes, which is also a known cause of proteotoxic stress in the matrix [105].

### Protein import stress in mammals

Not all the factors that combat mitochondrial import stress in yeast are conserved in higher eukaryotes, which complicates direct comparisons. Pdr3 and Rpn4, for example, have no obvious orthologs in humans, but a human protein with orthologous function to Rpn4 might be NRF1 [102], which is implicated in protein folding stress of the IMS of mitochondria (IMS-UPR) following insults through reactive oxygen species (ROS) [106]. Among many other effects, the NRF1-regulated transcriptional response also results in enhanced mitochondrial respiration and activation of the proteasome, similar to that of Rpn4 [107]. Yet, so far, no connection has been made between NRF1 and mPOS. In mammalian cell culture experiments, mPOS has been observed to result in the formation of aggresomes, a phenomenon which has not been reported for yeast cells [108]. Aggresomes are large spherical structures in the cytosol, that contain unfolded protein aggregates and that are formed when the UPS cannot keep up with degrading them [109]. Aggresome formation can, for instance, be triggered in HEK293T cells by overexpression of IMM carrier proteins beyond the import capacity of the TIM22 complex. The resulting structures contained not only the overexpressed hydrophobic carrier proteins but also other misfolded mitochondrial precursors. Additionally, a transcriptional response mediated by the immediate-early response gene EGR1 becomes activated [108]. EGR1 is rapidly activated following various stimuli, such as hypoxia, nutrient deprivation, cigarette smoke or mitogens, and—as one of many downstream events—induces autophagy-related genes [110]. In the context of mPOS, such genes might aid the sequestration of unfolded proteins into aggresomes [108].

Interestingly, aggresome formation was barely observed after import inhibition using chemical compounds (CCCP, MitoBloCK-6) or overexpression of matrix targeted proteins [108]. Therefore, the hydrophobicity of the accumulated carrier precursors seems to be the culprit of aggresome formation. In this study, the authors further differentiated between carrier-induced overaccumulation stress and misfolding stress. In addition to aggresome formation and autophagy activation, they found that the expression of a misfolded mutant of the carrier protein ANT1 (the mammalian ortholog of yeast Acc2) led to activation of genes involved in the UPS and the heat shock response [108]. Hence, parallels between expression of mutant versions of human ANT1 and its yeast ortholog can be drawn [13]. However, the formation of aggresomes distinguishes the human response to mPOS from the response in yeast. These structures spatially separate potentially dangerous unfolded proteins—some aggresomes are even membrane enveloped—from the cytoplasm and lower the burden on protein folding and mitochondrial protein import, indicating a protective role of aggresomes against mPOS in human cells.

If mPOS reflects the cytosolic consequence of mitochondrial import stress, what are the immediate effects on the mitochondrion itself? As the protein import machinery is central to the translocation of nuclear-encoded proteins, which make up ~ 99% of the mitochondrial proteome [8], it seems self-evident that almost every aspect of mitochondrial biology will at some point be affected by compromised protein import. The subunits of respiratory chain complexes, for example, are partially nuclear and partially mitochondrially encoded. Unless counteracted by the cell, an import defect of the nuclear-encoded subunits results in imbalances and potential aggregation of the now overabundant mitochondrially encoded subunits [105]. Besides dangers of aggregation, faulty assembly of respiratory chain complexes leads to inhibition of the electron transport chain, and in consequence decreased ATP synthesis, reduction of the membrane potential and ultimately an aggravation of the import defect [105]. In mammalian cells and nematodes, stress response mechanisms have been identified, that are activated by diverse perturbations, including mitochondrial import stress [111] (see below). However, how exactly the mammalian mitochondrion signals the import stress to the nucleus remains elusive. A mechanism resembling the yeast translocon salvage pathway for arrested precursors has also not yet been identified [14].

## Stress originating from the mitochondrial interior

### Mitochondrial stress triggers an unfolded protein response

Chaperones are molecular machines that guard the cellular proteome by keeping premature polypeptides in an unfolded state to aid the folding at the appropriate time and in the correct compartment. If a mitochondrial precursor is not captured quickly enough after its synthesis by the ribosome, misfolding errors can occur [112, 113]. To prevent the formation of toxic aggregates, a sub-class of chaperones, chaperonins, are able to locally unfold the misfolded parts of the protein and allow correct refolding [114]. Due to these essential functions, chaperones and chaperonins are constitutively expressed and can be found throughout the cell [115]. As described above, chaperones are required during the import of mitochondrial precursors in multiple capacities [37, 79–81]. Additionally, the mitochondrial matrix harbors a chaperonin complex consisting of HSP60 and HSP10 in humans, assisted by the mitochondrial HSP90 chaperone TRAP1, which ensures correct folding for the majority of mitochondrial matrix proteins upon conclusion of import [87, 116, 117].

While mitochondria are able to sense perturbations in their proteome, they do themselves not encode stress response genes and instead need to signal arising threats to the nucleus [3]. The discovery of such mito-nuclear retrograde flow of information in higher organisms raised questions about the different types of stress that are experienced and sensed by mitochondria and the nature of the pathways that relay these perturbations to the nucleus in these systems [19]. Initial experiments utilized depletion of mtDNA by ethidium bromide or overexpression of an aggregation-prone mutant of the matrix protein ornithine transcarbamylase (OTCΔ) to induce proteotoxic stress in mitochondria of rat hepatoma cells, which resulted in the activation of the UPRmt [18, 19]. However, it was suspected that unrelated types of perturbation of mitochondrial function could trigger a similar response if they exceed a certain threshold. Indeed, a diverse array of mitochondrial insults that affect the mitochondrial proteome and induce the UPRmt were subsequently validated experimentally. These include perturbations of mitochondrial translation by knockdown of mitochondrial ribosomes or treatment with doxycycline [105]. Moreover, insults that target mitochondrial protein quality control factors and decrease the folding capacity, such as knockdown or inhibition of mitochondrial proteases and chaperones ultimately causing proteotoxic stress, can also induce the UPRmt [118, 119]. Disruption of the electron transport chain is often used to induce UPRmt, either through depletion of individual nuclear-encoded components leading to a decrease in respiration and induction of mito-nuclear protein imbalance [120], or by inhibition of respiratory chain complexes with microbial toxins such as antimycin or oligomycin [121, 122]. The mitochondrial proteome is also threatened by disruption of the mitochondrial import system or dissipation of the mitochondrial membrane potential, both of which can activate this nuclear transcriptional program in worms [20].

Although these insults dramatically differ at first glance, their downstream consequences are interconnected. Mito-nuclear imbalances result in a reduction of respiratory chain complexes, which in turn affects the membrane potential. The reduction in ∆Ψm then causes import defects, which aggravates mito-nuclear imbalances, creating a vicious cycle. This gives reason to suspect that the cell might have evolved to sense a common signal. A groundbreaking study in 2019 approached this question by systematic knockdown of *C. elegans* genes to identify regulators of the UPRmt [123]. As a readout, the authors used a transcriptional reporter of *hsp-6*, the mtHSP70 chaperone in worms. Among the targeted processes were known and previously unknown triggers of the UPRmt such as disruption of protein import and disruption of metabolic pathways such as OXPHOS, the TCA cycle or lipid catabolism. The authors worked out that disruption of a majority of mitochondrial processes directly or indirectly exerts an effect on membrane potential and, therefore, decreases protein import—the proposed unifying signal for UPRmt activation [123]. This theory would implicate that the kinetics of chaperone induction can be correlated with the type of process which is perturbed. More specifically, direct loss of ∆Ψm using ionophores should induce chaperones faster than indirect insults such as knockdown of OXPHOS components. It will be interesting to see whether this scenario can be supported by further biochemical studies. It also does not rule out the possibility that additional sensing mechanisms and responses may exist in worms and other systems.

### Signaling mitochondrial stress to the nucleus in *C. elegans*

In a seminal study utilizing genetic screens, the principal abilities to sense mitochondrial stress and transcriptionally activate the UPRmt in *C. elegans* were found to be encoded in one and the same protein: ATFS-1 is equipped with an MTS and a nuclear localization signal (NLS) and can thus in principle localize to either compartment. Under steady-state conditions, this basic leucine zipper domain (bZIP) transcription factor is transported into the mitochondrial matrix, where it is degraded by the Lon protease. In the face of mitochondrial dysfunction, however, ATFS-1 import into mitochondria becomes unproductive and it instead enters the nucleus where it orchestrates the induction of UPRmt genes [20, 124]. The coordinated expression of a set of effector genes ultimately enables mitochondrial recovery in response to a variety of perturbations. While this necessitates the activation of diverse transcripts, the core factors of this program are the chaperonins *hsp-6* and *hsp-60* (HSPA9 and HSPD1 in humans), which increase folding capacity [125]. Additional quality control factors include the protease *ymel-1*, which combats aggregated or misfolded proteins by degradation [20]. Metabolic rewiring, through upregulation of glycolysis factors, and downregulation of OXPHOS and TCA cycle components facilitates an alternative means of ATP synthesis during stress [20, 126]. To boost mitochondrial function, factors involved in mitochondrial protein import, protein synthesis and mitochondrial dynamics are also induced [127].

**Fig. 2:**
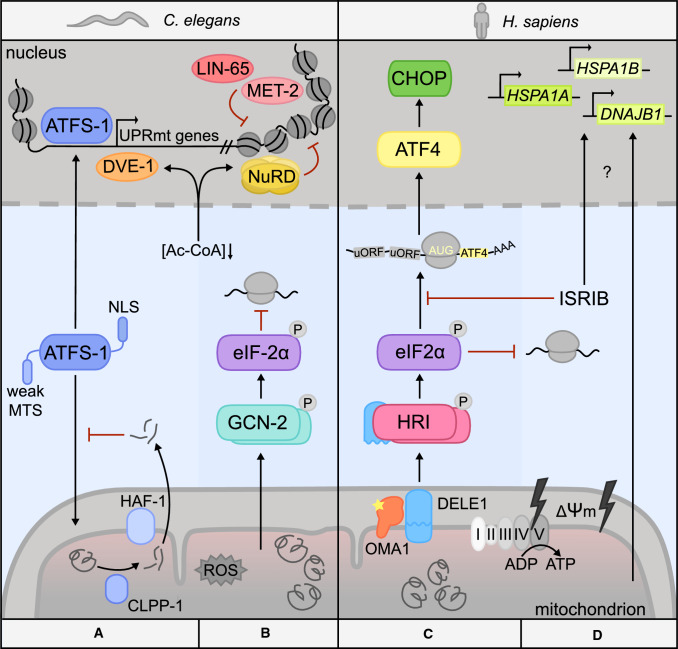
Principal response modules against stress originating from the mitochondrial interior in worms and humans. Differences and similarities in the response to mitochondrial dysfunction in *C. elegans* and *H. sapiens*. **A** UPRmt gene expression in worms is dependent on two stress signals: mitochondrial import efficiency and metabolic cues such as decreased acetyl coenzyme A (Ac-CoA) levels due to reduced mitochondrial TCA cycle activity. ATFS-1 acts as a sensor of mitochondrial import efficiency due to its weak MTS. Under conditions of perturbed import, ATFS-1 is imported into the nucleus via its NLS and activates transcription of UPRmt genes with the help of DVE-1 and its cofactor UBL-5. While histone demethylases JMJD-1.2/3.1 increase accessibility of UPRmt genes, LIN-65, MET-2 and NuRD complex members promote a global chromatin compaction through histone methylation and deacetylation and thus reduce the expression of other genes. Reduced TCA cycle activity during mitochondrial stress lowers cellular levels of Ac-CoA, which facilitates chromatin reorganization through nuclear recruitment of NuRD and DVE-1. **B** A cytoprotective translation attenuation is induced through GCN-2 activation and eIF2α phosphorylation. **C** In human cells, a diverse array of mitochondrial insults activates the mitoprotease OMA1, which in turn cleaves the mitochondria-resident factor DELE1. The C-terminal cleavage product (S-DELE1) subsequently accumulates in the cytosol, where it binds and activates the eIF2α kinase HRI. The resulting ISR signaling leads to a global attenuation of translation, while favoring expression of uORF-containing ISR master regulators like ATF4 and CHOP. **D** Unproductive ISR signaling in the context of mitochondrial stress because of genetic deficiencies in DELE1, HRI or pharmacological manipulation of eIF2B (ISRIB) results in the activation of a program dominated by heat shock protein expression

Besides stresses that immediately blunt mitochondrial import like dissipation of the membrane potential, ATFS-1 is also sensitive to sources of stress that more gradually interfere with its normal subcellular sorting. In *C. elegans*, aggregated matrix proteins are processed into peptides by the ATP-dependent protease CLPP-1 and then transported across the IMM by the ABC transporter HAF-1. Export of these peptides was proposed to affect mitochondrial import due to the effect of their charge on ∆Ψm, which results in relocalization of ATFS-1 to the nucleus (Fig. 2A) [123, 124, 128].

Induction of the UPRmt in nematodes is not only mediated by the subcellular partitioning of ATFS-1, but also involves extensive chromatin remodeling. The homeobox domain transcription factor DVE-1 and its co-activator UBL-5 bind to UPRmt effector genes to facilitate ATFS-1 recruitment and transcription of these loci [128, 129]. The chromatin of these genetic regions is rendered accessible by the activity of H3K27 demethylases JMJD-1.2 and JMJD-3.1 [130]. At the same time, several mechanisms reduce the transcription of other genes and implement a state of global chromatin compaction. LIN-65 and MET-2 promote gene silencing through histone methylation [131] and chromatin condensation is additionally advanced by a widespread reduction in histone acetylation. Recently, it was shown that mitochondrial stress induces nuclear accumulation of the nucleosome remodeling and histone deacetylase (NuRD) complex, which possibly coordinates histone deacetylation with the activities of LIN-65 and MET-2 [132]. At the same time, the histone deacetylase and NuRD component HAD-1 was found to interact with DVE-1 to induce expression of UPRmt target genes [133]. NuRD seems to be required for the efficient nuclear recruitment of DVE-1. Interestingly, in this setting, nuclear localization of NuRD and DVE-1 results from diminished acetyl-CoA levels due to reduced mitochondrial TCA cycle activity, underlining the importance of metabolic cues for UPRmt signaling [132]. Taken together, efficient activation of UPRmt effector genes and repression of other parts of the genome is not only dependent on mito-nuclear signal transduction by the stress-sensor ATFS-1, but also on large-scale chromatin remodeling events, that can be tuned by metabolic states. Since mitochondrial stress signaling during development in *C. elegans* has been associated with lifespan expansion due to early chromatin remodeling facilitating gene expression later in life [130, 131], it will be important to see whether these insights could be exploited to similarly counter some effects of aging in the human system.

### The integrated stress response

Apart from the canonical UPRmt signaling axis aimed at restoring proteostasis by increasing folding capacity and protease expression, mitochondrial stress also leads to a reduction of protein synthesis at the level of translation in *C. elegans*, further alleviating the pressure on the mitochondrial proteome. This is facilitated by the activation of a cellular program termed the integrated stress response (ISR) [134]. The ISR is a conserved response that can be triggered by diverse environmental stimuli, through one of four eIF2α kinases that phosphorylate the α-subunit of eukaryotic translation initiation factor 2 (eIF2α) at serine 51 [24]. This phosphorylation counteracts formation of the ternary complex consisting of eIF2, GTP and a methionyl-initiator tRNA, which comprises a crucial step in 5’cap-dependent translation initiation [135]. ISR activation leads to two protective adaptations in the cell: first, attenuation of protein translation due to reduced availability of ternary complexes alleviates the pressure on protein maturation and processing machineries and gives the cell the chance to restore the perturbed proteome [20, 136]. Second, preferential expression of select genes encompassing an upstream open reading frame (uORF), such as the transcription factors ATF4 and CHOP, ensures the subsequent generation of factors that actively aid cellular recovery [137] or, if appropriate, initiate apoptosis [138]. Dephosphorylation of eIF2α terminates the translational brake and is critical for the cellular fate after ISR signaling [139–142]. This is underlined by the embryonic lethality of mice double-deficient in the responsible phosphatases CReP and GADD34 [143].

The kinases mediating the central step of eIF2α phosphorylation in the ISR signaling pathway in mammalian cells are heme-regulated inhibitor (HRI), protein kinase RNA-activated (PKR), PKR-like ER kinase (PERK), and general control non-derepressible 2 (GCN2). While they share the necessity for dimerization and trans-autophosphorylation to become active and show substantial homology in their kinase domains, selective activation is controlled by their subcellular localization and regulatory domains [24]. What are the signals that trigger the respective eIF2α kinase?

The ER-resident kinase PERK is activated by unfolded proteins in the ER. The mechanism involves the HSP70 chaperone BiP and elegantly couples the degree of PERK activation to the abundance of unfolded ER proteins [144–147]. PKR is predominantly cytoplasmic but can also be found in the nucleolus and nucleoplasm. It is activated by double stranded RNA, which is often encountered in the course of viral infection [148, 149]. Additionally, PKR has been implicated in the response to oxidative stress, growth factor deprivation and Toll-like receptor activation [150].

GCN-2 has been shown to be activated in worms, when mitochondrial dysfunction is induced by ROS (Fig. 2B). This signaling does not require ATFS-1 and HAF-1 and, therefore, represents a separate cellular response to mitochondrial dysfunction. Through RNAi experiments, it was demonstrated that eIF2α phosphorylation by GCN-2 was increased when ATFS-1 was depleted during mitochondrial stress. Conversely, knockdown of GCN-2 increased chaperone expression under these conditions [151]. However, to which extent the respective pathways contribute to dealing with different kinds and intensities of stress and whether this involves crosstalk is currently unknown. Although the role of sensing mitochondrial stress has been assigned to GCN-2 specifically in *C. elegans*, a general, conserved function of GCN-2 lies in its ability to sense amino acid deprivation and oxidative stress, which allows conservation of nutrients and energy [152, 153]. The activation was proposed to be mediated through binding of deacetylated tRNAs, however, a recent report suggests that the kinase binds to the ribosomal P-stalk, which induces a conformational change and downstream signaling [154, 155].

HRI was initially found to function in erythropoiesis, where it senses changes in heme availability through its N-terminal domain, which keeps hemin-bound HRI in an inactivate state [156]. This coordination is needed for hemoglobin synthesis, because heme and globins are required in a stoichiometric ratio [157] and might be exploitable for the treatment of hemoglobinopathies [158]. Excessive production of alpha and beta globin can be detrimental through aggregation and induction of proteotoxic stress [159]. A more general role for HRI in cytosolic protein homeostasis beyond heme can be rationalized by its interplay with heat shock factors, including HSP90, HSPA8 and HSPB8 [160–162]. This led to the hypothesis that HRI functions in a cytosolic unfolded protein response (cUPR) that can be triggered by inhibition of the UPS or by large protein aggregates like such formed by α-synuclein and several innate immune signalosomes [160, 163]. These signaling platforms, also known as SMOCs (supramolecular organizing centers), are for instance utilized during antiviral signaling of MAVS at the mitochondrial surface [164] or peptidoglycan sensing via NOD1/2, and seem to involve HRI [160]. They were shown to sequester HSPB8 away from HRI, which represses the kinase when bound to it. Liberated HRI is then able to induce an ISR, which increases HSPB8 levels, improving signalosome stability and sustained immune signaling [160]. By extension, HRI might be similarly activated by pathological aggregates in the cytosol. It was found that knockdown of HRI enhances cytotoxic accumulation of overexpressed α-synuclein and that HRI-deficient mice display signs of α-synuclein misfolding. HRI seems to have a role in the autophagic clearance of such cytosolic protein aggregates and this activity was proposed to also apply to other toxic aggregates [165].

Whether additional stress kinases exist is an ongoing debate [166]. However, vastly different types of stress seem to be able to converge on one and the same eIF2α kinase, which highlights that contextual cues and upstream modulators may hold the key to a comprehensive understanding of ISR activation, signaling and translational response. As described above, the key downstream effects entail the global attenuation of translation and the expression of stress-responsive genes via the bZIP transcription factors ATF4, ATF5 and CHOP [24]. Stress-selective translation of these ISR master regulators is controlled by inhibitory uORFs. The main ORF of mammalian ATF4 is preceded by two such elements, whereas CHOP contains a single uORF [167, 168]. During 5’cap-dependent translation, the first start codon of such mRNAs is recognized by a functional translation initiation complex. In the context of ISR activation, availability of ternary complexes is reduced due to eIF2α phosphorylation, rendering assembly of functional translation initiation complexes less efficient. Consequently, a portion of already assembled ribosomes—waiting for a new ternary complex to initiate translation—spend more time on scanning the mRNA, eventually skipping the uORF start codon in favor of the start codon of the downstream primary open reading frame that encodes the stress response gene [135, 167]. In particular ATF4 and its downstream effector CHOP have been found to be essential for the response to different types of cellular stress, including mitochondrial perturbations like loss of ∆Ψm, inhibition of OXPHOS or mitochondrial translation and import defects [21, 23, 169]. While expression of ATF4 and CHOP appear to be central, how the ISR is modulated and tuned to the nature of the experienced stress is less well understood. CHOP acts by heterodimerization with a member of the C/EBP proteins [18, 170]. This leads to inhibition of the activity of the C/EBP protein and transcriptional induction of effector genes characterized by a CHOP responsive element in their promotor region [171]. Specific induction of effector genes upon mitochondrial stress seems to also require binding of AP-1 [172, 173]. Two additional regulatory elements have been proposed to further increase specificity: MURE1 and MURE2, however, a corresponding transcription factor has not yet been identified [170].

Differential activation of effector gene subsets has also been connected with a distinct cellular outcome: pronounced activation of ATF5 or CHOP has, for instance, been associated with pro-apoptotic signaling, suggesting an abortive response to overwhelming cellular perturbation [138]. Depending on its heterodimerization state, CHOP can function both as a transcriptional activator or a transcriptional repressor [174]. Among anti-apoptotic proteins repressed by CHOP are BCL-XL, BCL2 [175] and MCL1 [176]. Pro-apoptotic proteins induced by CHOP include TRB3 [177], BIM [178] and ERO1α [179]. Its induction of the phosphatase GADD34, which removes the phosphorylation on eIF2α and thereby the translational brake of the ISR, can also promote cell death if the cell did not yet manage to restore proteostasis [179]. In contrast, ATF3, which was shown to be activated in response to nutrient deprivation, although not in response to ER and mitochondrial stress, has been suggested to play a more protective role [21, 180]. As ISR signaling is implicated to play a role in a variety of diseases [181], being able to better pinpoint distinct cellular outcomes based on particular transcriptional co-regulators and effector gene patterns could render them useful biomarkers in certain contexts of human disease.

### The role of the ISR in mitochondrial stress in humans

While induction of *hsp-6* and *hsp-60* [119] as a consequence of ATFS1-mediated canonical UPRmt signaling has been firmly established in *C. elegans*, how mitochondrial stress is relayed to the nucleus in the mammalian system remained elusive for decades. ATF5 has similarities to ATFS-1, like a putative MTS, an NLS and a bZIP domain, and has been proposed as a functional ortholog as it can rescue effector gene induction in ATFS-1 depleted worms, arguing that the transcriptional regulation is conserved from worms to mammals [182]. At the same time, ISR induction mediated by ATF4 (and its downstream effector CHOP) was observed as the dominant reaction in mammals across diverse mitochondrial insults [23, 183]. Of note, similar to ATF4 and CHOP, the mRNA of ATF5 also contains a uORF and is thus preferentially translated in the context of ISR signaling [182, 184].

Using genome-wide forward genetic screens, in 2020, it was discovered that the ISR is unexpectedly activated by the stress kinase HRI in response to a broad range of mitochondrial insults, including disruption of the mitochondrial membrane potential, inhibition of the respiratory chain and perturbation of the mitochondrial proteome [21, 22]. These experiments also revealed that these types of stress initially alert the metalloendopeptidase OMA1 to cleave a previously little-studied mitochondrial protein named DELE1.

OMA1 is a protease of the IMM with a distinct architecture from the AAA proteases of the IMS and matrix and whose catalytic domain faces the IMS [185]. Although incompletely understood, maturation and activation of OMA1 are thought to involve autocatalytic cleavage events. Active OMA1 functions in an ATP-independent manner, however, due to its own degradation the time in which it can fulfill its catalytic function is limited [186, 187]. Among its well-known substrates is OPA1, which is also cleaved by the i-AAA protease YME1L1 [186, 188, 189]. Due to its role in mitochondrial fusion, OPA1 cleavage affects mitochondrial dynamics and results in mitochondrial fragmentation [190]. YME1L1 and OMA1 are both stress-sensitive proteases and a recent study showed that they can reciprocally degrade one another in response to distinct types of stress, thereby adding another layer of regulation [191]. Furthermore, OMA1 has been described to affect stability of respiratory chain complexes and its absence, therefore, leads to respiratory decline in yeast, zebrafish and mouse embryonic fibroblasts [192]. Due to the variety of molecular pathways in which OMA1 is involved, it is not surprising that its loss or deregulation is involved in a multitude of diseases [193–198]. While the precise identity of the signals that spark its proteolytic activity is an ongoing debate [186–188, 199, 200], it is clear that OMA1 has to be tightly controlled, given its connection with multiple critical cellular pathways.

The cellular role of DELE1 had gone unnoticed for the longest time, although a prior report linked it to apoptosis [201]. Upon apoptosis induction, DELE1 acts upstream of CASP3, CASP8 and CASP9 activation and has been reported to bind to death receptors together with DAP3. Conversely, DELE1 knockdown significantly suppresses caspase activation and increases viability [201]. DELE1 possesses an extended MTS upstream of seven tetratricopeptide repeat (TPR) motifs [201]. TPR domains consist of repeats of ~ 34-amino acid motifs that fold in a helix-turn-helix conformation resulting in versatile three-dimensional structures suitable for protein–protein interactions [202]. Cleavage of DELE1 by OMA1 in response to mitochondrial stress has been shown to produce the signal that alerts the cytosol of an ongoing mitochondrial insult and activates the ISR in an HRI-dependent manner [21, 22]. OMA1 cleaves full-length DELE1 (L-DELE1) near histidine 142 [22] to produce a shorter C-terminal fragment, S-DELE1, containing the TPR domains. Cleavage results in an accumulation of S-DELE1 in the cytosol, where it physically associates with the eIF2α kinase HRI and stimulates its activity (Fig. 2C). The required dimerization and autophosphorylation of HRI [203] is likely assisted by the TPR segments of DELE1, as revealed by deletion mutants. Despite its role in heme sensing, DELE1-stimulated activation of HRI seems to be independent of cellular heme levels [21, 22]. This raises the possibility that instead of heme, DELE1 might compete with factors like HSPA8 for HRI binding, possibly relieving its inhibitory effect on kinase activation [162]. The requirement for the OMA1-DELE1-HRI signaling axis in ISR activation downstream of mitochondrial insults is further underlined by the observation that a deficiency in DELE1 or HRI phenocopies the effects observed with the ISR inhibitor ISRIB [21], which binds to eIF2B and enhances ternary complex formation also during eIF2α phosphorylation [204, 205]. In contrast to ISRIB or HRI deficiency, OMA1 and DELE1 represent putative points of intervention that are selective for mitochondrial stress over other cellular ISR triggers.

In addition to the ISR, an induction of the mitochondrial chaperones HSPD1 and HSPE1—reminiscent of UPRmt elements in worms—has also been observed under certain conditions of proteotoxic mitochondrial stress in HeLa cells [118]. Interestingly, in cells defective for the DELE1 signaling axis, upregulation of heat shock proteins was observed, suggesting the existence of an alternative response to mitochondrial dysfunction also in the human system [21, 22] (Fig. 2D). This is reminiscent of a similar finding in *C. elegans*, where inhibition of mitochondrial proteostasis factor *hsp-6* led to induction of the cytosolic heat shock response [206]. It was proposed that this response results in buffering of proteotoxicity from the mitochondria and is facilitated by changes in lipid biosynthesis. The authors suggest that there is a complex interplay between the different cellular UPRs and that a defective UPR branch is sensed by other stress pathways through metabolic changes [206]. Given these parallels, it will be of interest to identify how the observed heat shock response is mechanistically elicited in mammalian cells.

### Stress signal propagation between organelles and cells

Besides this core mitochondrial stress relay, additional inter-organelle crosstalk seems to be involved in the cellular stress response mechanisms: during ER stress, activation of PERK leads to increased expression of mitochondrial proteostasis factors such as LONP to counteract mitochondrial dysfunction through translation inhibition due to eIF2α phosphorylation [207]. Additional evidence for ER-mitochondria stress crosstalk comes from the recent finding that induction of the unfolded protein response in the ER triggers a metabolic re-programming of mitochondria towards increased 1C metabolism [208]. In light of the extensive physical and functional connectivity between the ER and mitochondria, especially at mitochondria-associated ER membranes [209], it will be of interest to also explore direct reciprocal effects the organelles may exert on one another in the context of perturbation and clarify the potential impact on other interacting organelles.

Stress signaling is not limited to relays between organelles but can also be propagated within tissues and throughout the organism. A landmark study in *C. elegans* demonstrated that mitochondrial perturbation in neurons leads to activation of UPRmt in cells and tissues that have not experienced the initial stress, indicating the existence of a diffusible cytokine-like factor (‘mitokine’) that infers stress resistance and increased survival [120]. Similar crosstalk was later reported between the worm germline and intestine [210]. Depending on the UPRmt model, activation of UPRmt effector genes in peripheral tissues can involve the short-range active neuropeptide FLP-2 which is induced upon ATFS-1 signaling, as well as the neurotransmitter serotonin [211, 212]. In a recent systematic approach to identify any long-range mitokines and associated signaling pathways, worms were chemically mutagenized and screened for deficiencies specifically in the cell non-autonomous mitochondrial stress response. This uncovered retromer-dependent Wnt signaling as a long-range mediator of the UPRmt alongside serotonin between the nervous and intestinal systems [213]. It will be important to determine whether this mechanism is conserved in the mammalian system, where beneficial effects of serotonin on mitochondria have been observed in some settings [214, 215]. In mammals, two diffusible factors, fibroblast growth factor 21 (FGF21) and growth/differentiation factor 15 (GDF15), have been found to be induced upon mitochondrial dysfunction and signal into distant tissues [216, 217]. Expression of FGF21 has been shown to be triggered in patients and mouse models with mtDNA mutations [218, 219] and consequentially leads to changes in lipid and energy metabolism [217]. GDF15 has also been reported to be upregulated in patients suffering from mitochondrial disorders and suggested as a biomarker for these diseases [220]. A possible metabolic component may be supported by the effect GDF15 exerts on appetite [221, 222], also mirrored in the reduction in inflammation, which is observed in mice overexpressing GDF15, and possibly stems from lower amounts of adipose tissue [223]. Upregulation of GDF15 in response to pathogenic stimuli also results in metabolic changes that promote survival and tissue protection [224], raising the question whether such phenotypes also involve mitochondrial stress signaling. A role for the UPRmt in innate immune responses has been observed in multiple settings. Microbial pathogens have been shown to induce mitochondrial dysfunction in *C. elegans,* resulting in UPRmt activation and expression of innate immune factors such as secreted lysozyme and anti-microbial peptides. Concomitantly, UPRmt activation is crucial for pathogen clearance and survival [225]. Interestingly, recent studies point out that metabolic enzymes and intermediates in the host and the pathogen can influence UPRmt induction during infection [226, 227]. These and related scenarios underline that mitochondrial stress signaling exceeds a locally limited response and instead can exert effects throughout the organism and its microbial invaders.

## Mitochondrial degradation

### Sacrificing mitochondria in an autophagic cascade

The aforementioned quality control pathways aim at recovery of the mitochondrial network upon stress by bolstering folding capacity and transiently reducing the burden of newly synthesized mitochondrial proteins. Under certain circumstances, those mechanisms are not sufficient to cope with the damage. Severe oxidative stress, hypoxia, collapse of ∆Ψm and aggregation of unfolded proteins can irreversibly disturb the mitochondrial proteomic integrity [25, 228]. As mitochondria are highly dynamic and constantly undergo fusion and fission, proteotoxic stress can propagate throughout the network and jeopardize overall organelle functioning, potentially resulting in collapse of cellular respiration and ATP insufficiency. Disturbed proteostasis can also result in OXPHOS malfunction and excessive ROS generation [229]. Increased ROS levels not only damage mtDNA [230] but also interfere with additional cellular functions [231]. To prevent a grave disturbance of cellular bioenergetics, cell death or disease, the organelle needs to be partially sacrificed. The irreparable parts are recognized, selectively segregated from the mitochondrial network and degraded by a specific autophagy mechanism termed mitophagy [232–234]. This process was initially discovered in yeast, where it was observed that mitochondrial fragments localize in the vacuole [235]. Mitophagy initiation in yeast involves proteins from the autophagy-related gene family (Atg) which localize to mitochondria and are not essential for bulk autophagy. Mitophagy requires the formation of an isolation membrane and the engulfment of designated mitochondria into an autophagosome [236]. This is accomplished by the recruitment of mitophagy receptors to the surface of damaged mitochondria, which bridge the mitochondrial cargo and the autophagosome machinery. In yeast, the specific degradation of mitochondria is regulated by the mitophagy receptor Atg32 [237]. Atg32 interacts with adaptor proteins Atg8 [238], which localizes at the isolation membranes, and Atg11 [239], which results in recruitment of the cargo to the phagophore assembly sites. In mammals, the process is conserved and the phagophore assembly factors are recruited by mitophagy receptors through interactions with LC3 or GABARAP family members, which are orthologous to yeast Atg8 [240, 241]. LC3/GABARAP are crucial for the engulfment of the dysfunctional organelle into autophagosomes, transport to and subsequent fusion with the lysosome, where the mitochondrion is ultimately degraded. Although all mitophagy receptors share a LIR (LC3 interacting region) motif and, therefore, can directly interact with LC3/GABARAP, autophagosomal membranes can also be recruited in an LC3/GABARAP-independent manner. Upon knockout of all LC3/GABARAP proteins, autophagosomes can still be selectively formed around damaged mitochondria [242]. This is rationalized by the observation that ubiquitinated mitochondrial proteins can recruit the mitophagy receptor NDP52, which in turn recruits the autophagy-initiating unc-51-like-kinase ULK1 for initiation of autophagosome formation without the help of LC3/GABARAP. Additional factors such as TANK-binding kinase 1 (TBK1) and FIP200 are necessary for ULK1 complex formation [243]. These findings illustrate that receptor-mediated autophagosome formation can be facilitated by at least two mechanisms: direct interaction with preformed LC3/GABARAP phagophores via the LIR motif or recruitment of the ULK1 complex facilitating autophagosome assembly independently of LC3 proteins [242, 243]. However, after formation of autophagic bodies, LC3 and GABARAP are still required for the lysosomal fusion reaction [242, 244]. While the common downstream consequence of mitophagy is the lysosomal degradation of damaged mitochondria, its initiation can be separated into two major mechanisms: PINK1/Parkin-dependent and PINK1/Parkin-independent pathways.

### PINK1/Parkin-mediated mitophagy

**Fig. 3:**
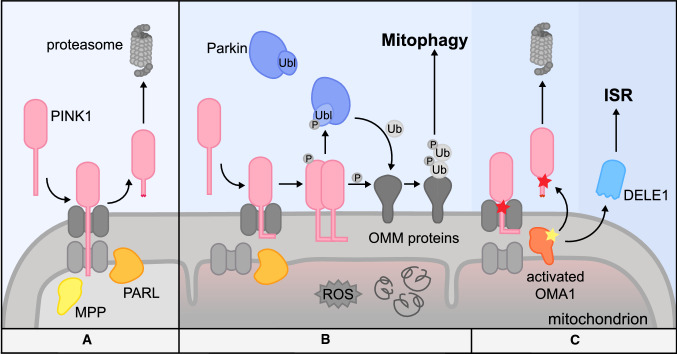
Regulation of the mitophagy factor PINK1 by different proteases. **A** Under homeostatic conditions, the N-terminus of PINK1 is imported through the TOM and TIM complex into the matrix where its MTS is cleaved by MPP. Additionally, PARL cleaves PINK1 at alanine 103 and the C-terminal fragment containing the kinase domain is retro-translocated to the cytosol for proteasomal degradation. **B** Severe mitochondrial stress stalls import across the IMM and disrupts PINK1 processing. This results in the stabilization of PINK1 on the OMM, presumably by lateral release from the TOM complex. Subsequently, PINK1 undergoes dimerization and activating autophosphorylation. Activated PINK1 phosphorylates ubiquitin entities on OMM proteins as well as a ubiquitin-like domain in Parkin. This results in a positive feedback reaction of PINK1/Parkin-dependent phospho-ubiquitination on the mitochondrial surface. OMM proteins modified in this manner recruit mitophagy receptors that coordinate the destruction of the organelle in lysosomes. **C** Certain PD-related PINK1 mutants fail to be stabilized on the OMM in response to mitochondrial stress. Instead, they are processed by OMA1, the mitoprotease which is also involved in the cleavage of DELE1 in ISR induction

PINK1/Parkin-dependent mitophagy is a vast field of research and we will limit its discussion to the essential aspects and refer to other excellent reviews for further detail [245, 246]. Initiation of this pathway relies on two proteins that act together: PTEN-induced kinase 1 (PINK1), a sensor for mitochondrial dysfunction and its interaction partner, the E3 ubiquitin ligase Parkin [247, 248]. Mutations in *PINK1* or the Parkin-encoding gene *PRKN* are associated with autosomal recessive, early-onset forms of Parkinson’s disease (PD) [249, 250]. Similar to the roles of ATFS-1 and DELE1 in the coordination of the UPRmt and ISR signaling, respectively, PINK1 acts as a sensor of mitochondrial damage that can initiate mitophagy. PINK1 is constitutively expressed and is targeted to mitochondria by its MTS, where import occurs via the TOM and TIM complexes [251]. During cellular homeostasis, PINK1 is a client of two mitochondrial proteases: MPP, which removes the presequence, and IMM resident protease Presenilins-associated rhomboid-like protein (PARL), which cleaves downstream of the MTS at the position alanine 103 [252, 253]. PARL cleavage destabilizes PINK1 as it results in its retro-translocation to the cytosol for proteasomal degradation (Fig. 3A) [251, 254]. While DELE1 accumulates in the cytosol upon mitochondrial perturbation, unprocessed PINK1 is stabilized on the OMM in response to mitochondrial insults [21, 247, 255]. How PINK1 is released from the TOM complex into the OMM in this case is not well understood, but the mechanism seems to involve TOM7 [256]. Stabilized full-length PINK1 subsequently initiates the downstream multistep mitophagy program [247]. Thus, import failure and stabilization of PINK1 correspond to the stress-sensing function of the kinase.

In a recent study, OMA1 was identified as an additional mitoprotease that can affect PINK1 stability. It was found that certain PD-associated PINK1 mutants fail to accumulate on the OMM and are degraded in an OMA1-dependent manner (Fig. 3C) [256]. While it is unknown whether OMA1 also plays a role in the life cycle of wildtype PINK1 under specific circumstances, this finding might be of significance for potential future therapeutic approaches in the context of certain mutant variants of the protein.

After stabilization on the OMM and activating autophosphorylation [257], PINK1 phosphorylates two major substrates: ubiquitin found on OMM proteins [258–260] and the ubiquitin-like domain (Ubl) of Parkin, both at serine 65 [247, 261]. Ubl phosphorylation of Parkin activates its ubiquitin ligase activity [262]. Phosphorylation of ubiquitin serves as a recruitment signal for additional Parkin to the mitochondrial surface, which is in turn also phosphorylated by PINK1. Together, this creates a positive feedback that results in massive ubiquitination and phosphorylation of OMM substrate proteins (Fig. 3B) [262, 263]. While phospho-ubiquitin is essentially undetectable on unperturbed mitochondria, it rapidly accumulates to nearly 20% of the total ubiquitin after mitochondrial depolarization [264].

Although PINK1 positively regulates mitophagy, recently, it has been assigned a self-antagonizing role by phosphorylating the non-canonical mitochondria-cytosol dual localized translation elongation factor TUFm. TUFm supports Parkin recruitment and promotes mitophagy when localized on mitochondria independently of PINK1. Overexpression of TUFm in a PINK1 knockout background is sufficient to restore mitophagy. Upon mitophagy initiation, however, stabilized PINK1 phosphorylates mitochondrial TUFm at serine 222 resulting in its cytosolic relocalization where it interferes with ATG5–ATG12 conjugation and thus suppresses mitophagy. The proposed mechanism suggests that PINK1 can buffer its activity resulting in suppression of excessive mitophagy [265]. Furthermore, several mitochondrial E3 ligases fine-tune Parkin activation and regulation: MUL1 suppresses Parkin recruitment to the OMM by maintaining ER-contact sites and MITOL ubiquitinates OMM proteins to increase Parkin activation by priming the mitochondria for PINK1/Parkin-dependent mitophagy [266, 267]. By modulating the abundance of pre-installed ubiquitin entities on OMM proteins, MITOL may govern the rate of mitophagy.

Parkin acts as an E3 ubiquitin ligase for at least 21 OMM proteins, including VDAC, MFN1 and MFN2 [268–271]. It was shown that Parkin is able to assemble canonical and non-canonical ubiquitin chains in vivo, most prominently K48- and K63-linked followed by K6- and K11-linked chains. Interestingly, in vitro the K6- and K11-linked ubiquitin conjugates appear to dominate over K48- and K63-linked chain types [264], although the implication of this observation remains to be elucidated. Under basal conditions, Parkin is auto-ubiquitinating itself by forming K6-linked ubiquitin chains which serve an inhibitory function. USP8, a deubiquitinating enzyme (DUB), has been shown to counteract this activity by removing K6-linked ubiquitin conjugates from Parkin and thus positively regulate mitophagy [272]. In contrast to USP8, the DUBs USP30 and USP15 have been assigned roles in dampening mitophagy. USP30 is mitochondrially anchored [273] and deubiquitinates OMM localized Parkin substrates, preferably removing K6- and K11-linked chains [274]. Overexpression of USP30 thus counteracts mitophagy in response to IMM depolarization, whereas its knockdown stimulates mitophagy initiation [275]. Since USP30 has been recognized as a Parkin substrate itself, it is speculated that unleashed Parkin can override these inhibitory effects on mitophagy by destining USP30 for degradation [275]. Similar regulatory effects on mitophagy have been ascribed to the cytosolic DUB USP15 [276]. The discovery of this type of interplay has fueled the development of DUB inhibitors as a means of boosting mitophagy, as this is expected to be beneficial in the context of PD and related diseases [277]. OMM proteins, which are poly-ubiquitinated by Parkin and phosphorylated by PINK1 serve as a platform for autophagy receptor recruitment. The primary receptors are CALCOCO2 (NDP52) and Optineurin (OPTN) [278]. Additional receptors include SQSTM1 (p62) [240], NBR1 [279] and TAX1BP1 [280]. Recently, the band of mitophagy receptors received an unexpected addition with Prohibitin 2 (PHB2), which localizes to the IMM rather than the mitochondrial surface. This led to the hypothesis that this IMM receptor may become exposed after Parkin-mediated proteasome-dependent OMM rupture [281, 282]. Additionally, it was proposed that PHB2 could aid in the stabilization of PINK1 on the OMM after mitochondrial depolarization by negatively regulating PARL [283].

### Mitophagy pathways that do not require Parkin

Beyond the canonical PINK1/Parkin axis, mitophagy can also be executed by independent mechanisms. This type of mitophagy relies on receptor-dependent recruitment of the autophagosome components directly to the damaged organelle. Although these receptors share functional similarities with PINK1/Parkin-dependent receptors, they do not require ubiquitin chain formation for their recruitment. These receptors include Bcl2 like protein 13 (BCL2L13) [284], a mammalian homolog of the yeast mitophagy receptor Atg32, FK506 binding protein 8 (FKBP8) [285], FUN14 domain containing 1 (FUNDC1) [286], BH3-only Bcl-2 family protein (BNIP3) [287] and its homolog Nip3-like protein (NIX/BNIP3L) [288], nucleotide-binding domain and leucine-rich repeat–containing protein X1 (NLRX1) [289] and Autophagy and Beclin 1 Regulator 1 (AMBRA1) [290]. These receptors differ in their activating trigger, their interaction partners and their involvement in physiological processes. For instance, BCL2L13, has been shown to interact with ULK1 to recruit the autophagosome machinery [284, 291]. FKBP8 has been reported to respond to depolarization [285], whereas FUNDC1, BNIP3 and NIX/BNIP3L are activated during hypoxia [286–288, 292, 293]. NLRX1 acts in pathogen-induced mitophagy to promote survival during microbial and viral infection and functions as an anti-inflammatory regulator in macrophages [289, 294, 295]. AMBRA1 interacts with HUWE1 which induces ubiquitination of MCL1 and MFN2 [290, 296], creating a possible node for crosstalk with PINK1/Parkin signaling.

The existence of multiple, possibly redundant pathways for mitochondrial clearance not only underlines the importance of this cellular stress response mechanism, but also raises questions with respect to their physiological activation. Does the cell select between pathways that respond to identical stimuli or activate those in parallel [287, 288, 292]? Growing evidence points at careful coordination between the pathways. For instance, certain receptors such as NIX can act in PINK1/Parkin-dependent and -independent pathways, supporting the possibility of a multipronged mitophagic response [297, 298].

## Impact of mitochondrial dysfunction on lifespan and disease

Mitochondrial function and proteostasis are tightly intertwined and their perturbation has been associated with aging, neurodegeneration and a variety of other diseases including myopathies and heart failure [1, 3]. Mitochondrial dysfunction is particularly detrimental in neurons and cardiomyocytes due to their increased energy demand needed to sustain excitability or contractility and their post-mitotic state [30, 31, 299]. The overarching aim of the mitochondrial stress response mechanisms described throughout the review is sensing and signaling mitochondrial damage to resolve the source of the stress to prevent further mitochondrial dysfunction. This has been shown to not only impact lifespan, but also healthspan and is, therefore, of particular biomedical interest.

In *C. elegans*, activation of UPRmt signaling in response to mild mitochondrial stress during development has been shown to positively impact lifespan [32, 120]. This is accomplished by extensive chromatin remodeling mediated by factors including the histone methyltransferase MET-2 and histone demethylases JMJD-1.2/JMJD-3.1 [130, 131], propagating a beneficial chromatin state with lifelong benefits. The discovery that the NuRD complex induces chromatin changes in response to metabolic changes that arise from mitochondrial dysfunction [132], reinforces the paradigm of dietary impacts on longevity [32, 300]. While the biology of mitochondrial stress in humans is less well understood than in yeast or worms, these data suggest that modulation of the functionally analogous pathways might in principle also be exploitable in human aging. Activation of mitophagy, too, can exert positive effects on longevity. In *C. elegans*, DCT-1 (the putative orthologue to the mammalian NIX/BNIP3L and BNIP3) acts downstream of PINK-1 in the turnover of damaged organelles and mitochondrial biogenesis. Its loss leads to an increase in mitochondrial mass alongside mitochondrial damage and reduces lifespan as a result of diminished stress resistance [301].

Beyond lifespan, it is also becoming increasingly clear that mitochondrial dysfunction significantly affects organismal healthspan. It has, for instance, been observed, that a UPRmt-like signature is induced in humans and mice in response to hypertension. Strikingly, bolstering the mitochondrial capacity to handle the hypertension-induced stress by pre-treatment with nicotinamide riboside [302], significantly reduces mitochondrial dysfunction and increases cardiomyocyte survival and contractility [303]. In Alzheimer’s disease (AD), accumulation of amyloid plaques and intracellular tau fibrils are commonly observed and might play a central role in pathogenesis through proteotoxicity [115]. Mitochondrial dysfunction, for instance detectable through computational analysis of AD patient brain expression datasets [302], has come into focus as a putative underlying cause of disease [31]. How this relates to the observed protein aggregates is an active field of research. Interestingly, it was shown that amyloidogenic peptides localize to mitochondria and can interfere with mitochondrial import [304]. Their accumulation disturbs the presequence processing capacity, which subsequently increases the amount of immature precursors in the organelle [305]. Another study found that Aβ, in particular the AD-associated Aβ42 variant, infers toxicity by aggregation with mitochondrial precursors in the cytosol, and further aggravates mitochondrial dysfunction [306]. An upregulation of mitochondrial stress response pathway genes (UPRmt and mitophagy) can be observed in nematode and mouse models of AD pathology, as well as in human patient data [302]. In the worm, mitochondrial defects are intensified when UPRmt is defective and increased UPRmt activation through inhibition of mitochondrial translation results in a reduction of Aβ aggregation. Similarly, in cultured human neuroblastoma cells, an ISR-dependent reduction of amyloid plaque formation following translation inhibition could be observed [302]. Although further insights into the mechanistic aspects of mitochondrial import interference by amyloid peptides are to be expected, these findings already indicate that boosting mitochondrial proteostasis through activation of mitochondrial stress responses might yield tangible clinical benefits.

Possible negative impacts of mitochondrial stress are not limited to dysfunction of the organelle itself, but aberrant firing of the otherwise cytoprotective stress signaling pathways can itself also become detrimental. Prolonged mild UPRmt activation has been shown to aid the propagation of mtDNA mutations, which aggravates the underlying cause of mitochondrial dysfunction [127, 307]. Similarly, the mitoprotease OMA1 appears to be involved in the destruction of certain PINK1 variants observed in PD [256]. Given its role in OPA1 processing and thus mitochondrial dynamics, inhibition of OMA1 is being explored as a therapeutic approach in the context of heart failure [194], neurodegeneration [196] and kidney ischemia reperfusion injury [198]. At the same time, OMA1 inhibition was found to increase proliferation and metastasis of breast cancer cells [195] and aggravate obesity, while impairing thermogenesis in mice [197]. In light of its newly discovered additional function in DELE1/HRI-mediated ISR signaling, it will be of interest to closely dissect the beneficial and adverse effects of this mitoprotease, and whether these might be separable by modulating its downstream effectors, such as DELE1, HRI, eIF2α, OPA1 or PINK1.

## Conclusion and perspectives

Safeguarding mitochondrial protein homeostasis is key for overall cellular health and depends on basal quality control factors as well as tunable stress response pathways [4]. Distinct properties of mitochondria, such as balancing gene expression of two genomes, protein import across two membranes, and the absence of mitochondria-encoded quality control mechanisms complicate this task [3].

As we elucidated in this review, import alone requires a complex machinery to ensure the arrival of functional proteins at the correct destination in the necessary quantity. We accentuated two layers of cellular responses at the mitochondrial surface that combat impaired protein import in yeast: (1) mitoTAD and mitoRQC, steady-state surveillance mechanisms that remove stalled precursors from the import pore [96] and (2) invocation of mito-nuclear defense modules including UPRam, and mitoCPR in the face of cytosolic precursor accumulation stress (mPOS)—a sign of a more substantial import defect [13, 14]. Importantly, it is much less clear how similar challenges surrounding the mitochondrial import pore are controlled in the mammalian system. Since cytosolic aggregates are a core feature of many neurodegenerative diseases, the discovery of corresponding stress response mechanisms designed to counteract accumulation of aggregation-prone mitochondrial precursors in mammalia could be of significant clinical impact [1]. HRI has been reported to be essential for the assembly of innate immunity signalosomes, which similarly pose a potential threat to the cell through uncontrolled aggregation [308, 309]. The assembly of α-synuclein containing fibrils in the cytosol (as observed in PD) also alters the cytosolic folding environment and triggers HRI-dependent ISR signaling: Heat shock proteins, which normally keep HRI in an inactive state, are recruited away from HRI to associate with the forming fibrils, which results in the expression of ATF4 and inflammatory cytokines [160]. It will be important to identify whether HRI can similarly respond to aggregation-prone precursors in the context of defective mitochondrial protein import.

Proteostatic control of the mitochondrial interior depends on separate quality control factors that include proteases and chaperones. As a response to protein folding stress inside of mitochondria, the UPRmt remodels the cellular proteome to bolster folding capacity and facilitate cellular recovery [4]. Besides this program, which has been most intensely studied in worms, a pathway consisting of OMA1, DELE1 and HRI that activates the ISR, was recently discovered as the predominant response in cultured human cells [21–23]. A common aspect of these mechanisms is the dependence on a mitochondria-targeted stress-sensor (ATFS-1 and DELE1) that is under the control of a mitochondrial protease (LONP and OMA1, respectively) and changes its subcellular localization in the face of mitochondrial perturbation (nuclear in the case of ATFS-1, cytoplasmic for S-DELE1). Both modules activate programs that can help to restore mitochondrial function, although by different means: as such, the core heat shock factors in UPRmt, HSP60 and HSP10, are not elicited by the ISR, whereas translational inhibition is not a principal outcome of UPRmt signaling. In worms, UPRmt and the ISR can be activated in parallel and similarly, in mammalian cells, expression of *HSPD1* and *HSPE1* (alongside related UPRmt signature genes in some instances [23]) was observed under certain settings of mitochondrial stress, including mtDNA deletion, ROS formation, or protein misfolding in the mitochondrial matrix [18, 19, 118, 182]. It will be of great interest to see whether additional signaling modules can be identified and how these interact in mammals. It also raises the possibility that tissue type and other contextual signals, such as the metabolic state, might have an impact on the relative weight of the respective branch of the response.

Certain mitochondrial insults are of a magnitude that warrants sacrificing parts of the organelle in mitophagy to prevent propagation of damage within the network and salvaging the biological building blocks. Although our understanding of PINK1/Parkin-dependent and -independent mitophagy pathways is continuously expanding, questions about the distinct triggers, co-dependencies and putative crosstalk remain. This is further complicated by potential differences between in vitro and in vivo situations. For PINK1 and Parkin, the commonly applied practice of Parkin overexpression in cultured cells and stimulation of mitophagy by severe mitochondrial insults that may not closely recapitulate physiological conditions has raised concerns [310, 311]. Accumulation of misfolded proteins in the mitochondrial matrix has been suggested as a more physiological context for the investigation of PINK1/Parkin-mediated mitophagy [255]. Careful measurements of basal mitophagy in vivo revealed that this organelle turnover does not seem to rely on PINK1 and Parkin and that their involvement may be restricted to certain high-stress triggers [312]. On the other hand, PD-associated mutations in PINK1 and Parkin argue in favor of a strong requirement for this pathway in coping with mitochondrial stress experienced in the course of human life [249, 250].

Considering this variety of protein homeostatic pathways, the question arises how the cell is able to launch an appropriate type of response (or combination thereof) to the encountered stress and tune its intensity. This decision-making needs to take into consideration questions of energy expenditure (as energy might be particularly limiting when mitochondria malfunction) and the ultimate outcome (recovery or programmed cell death) that best serves the organism. Given the multitude and in part seemingly overlapping functions of the mitochondrial safeguards, the hypothesis of a certain unifying stress signals has been brought forward [123]. For the UPRmt in *C. elegans*, the various triggers have been suggested to eventually converge on defective import and thus mito-nuclear redistribution of ATFS-1. Interestingly, although DELE1 also changes its localization upon activation, its efficient accumulation in the cytosol relies on OMA1-mediated cleavage [21, 22]. In contrast, subcellular redistribution of ATFS-1 seems to be mediated solely by its dual sorting signals [20]. In this, it also differs from the OMA1 substrate PINK1: whereas both factors are continuously degraded under steady-state conditions, unlike ATFS-1, the degradation of PINK1 takes place in the cytosol and thus requires retro-translocation from mitochondria [247]. This is not only reminiscent of the import pore surveillance mechanisms in yeast but also OMA1-mediated cleavage of DELE1. However, it relies on a different IMM protease—PARL. In the absence of PARL cleavage, wildtype PINK1 accumulates at the mitochondrial surface and can initiate mitophagy [251]. Of note, the discovery that certain PD-associated mutant versions of PINK1 can alternatively be cleaved by OMA1 [256] places this protease at the interface of DELE1-mediated ISR signaling and mitophagy. This further emphasizes the question of putative crosstalk between the different levels of mitochondrial stress response modules. Does retro-translocation of PINK1 involve mechanisms related to those guarding the import pore in yeast? Are PINK1 and DELE1 able to compete for cleavage by OMA1 and does this result in a state of aberrant ISR signaling in certain forms of PD beyond defects in the mitophagy cascade? Or does the detrimental effect of certain PD mutations alternatively perhaps not merely reflect a defect in mitophagy but in part result from an overburdening of the alternative stress response modules?

Beyond differential activation and putative crosstalk between the different mitochondrial stress response mechanisms, the intensity of the respective individual responses can also significantly alter the cellular outcome. Particularly activation of the ISR and mitophagy pose a potential threat to the survival of the cell and need to be tightly controlled: PINK1/Parkin-mediated mitophagy involves a runaway reaction that produces the autophagy signal phospho-ubiquitin [263] and needs to be constrained to the damaged sections of the mitochondrial network. Activation of the ISR grants time to cope with stress imposed by unfolded proteins but comes at the price of a dramatic reduction in overall cellular protein synthesis. Prolonged activation is thus incompatible with cellular survival [138]. As described, a basal level of housekeeping of the mitochondrial import pore can be escalated to more severe clearance of precursors in separate pathways via involvement of nuclear transcription cascades in yeast. For ATFS-1, integration of mitochondrial stress signaling at the level of import is thought to allow for a tuning of the transcriptional response to the severity of import problems. This is accomplished by the dual localization of ATFS-1 mediated by its weak MTS and NLS [123, 313]. The degree of a mitochondrial import defect thus correlates with nuclear levels of ATFS-1 and in turn the strength of the transcriptional response. Similarly, DUBs and the turnover of autophagy receptors tune the level of mitophagy [275, 314]. This raises the question whether the OMA1-DELE1-HRI axis can also be activated to different extents and how signaling is modulated in the context of different cellular insults. What would be the role of OMA1-mediated cleavage of DELE1 during mitochondrial import stress, particularly if this stress arises at the TOM complex? Does this result in the cytoplasmic accumulation of L-DELE1 and does this species contribute to the stress response in an mPOS-like fashion? If so, does this involve binding and activation of HRI as observed when the stress stems from the mitochondrial interior, or are ancillary mechanisms involved? It has, for instance, been proposed that DELE1 may have an additional effect on ATF4 that acts downstream of and is, therefore, independent of eIF2α phosphorylation, although the mechanics of this effect remain to be deciphered [22]. It is also conceivable that, depending on the nature of mitochondrial damage, productive ISR signaling requires additional components. Beyond the core pathway components, genome-wide analysis of factors required for the expression of CHOP in the context of mitochondrial depolarization revealed several auxiliary hits, including metabolic regulators and CLUH [21]—a protein involved in the control of the translation of mRNAs coding for mitochondrial proteins [315, 316] that has recently also been linked to the coordination of mitophagy [317]. Finally, pathway components could be subject to post-translational modifications that might attune downstream signaling to the nature and severity of the experienced mitochondrial insult. Proteomics and single cell approaches may help to shed further light on such aspects. It will also be of interest to extensively characterize variants of the discussed stress module components found in the human population in the context of mitochondrial fidelity. These and other experiments may reveal hitherto undisclosed layers of modulation of mitochondrial stress signaling in the human system.

## Data Availability

Not applicable.

## Abbreviations

∆Ψm: Mitochondrial inner membrane potential
Ac-CoA: Acetyl coenzyme A
AD: Alzheimer’s disease
bZIP: Basic leucine zipper domain
CCCP: Carbonyl cyanide *m*-chlorophenyl hydrazone
cUPR: Cytosolic unfolded protein response
DUB: Deubiquitinating enzyme
ER: Endoplasmic reticulum
HAP: Heme activator protein complex
IMM: Inner mitochondrial membrane
IMP: Inner membrane peptidase
IMS: Intermembrane space
IMS-UPR: Unfolded protein response in the mitochondrial intermembrane space
ISR: Integrated stress response
ISRIB: Integrated stress response inhibitor
LIR: LC3 interacting region
mitoCPR: Mitochondrial compromised protein import response
mitoTAD: Mitochondrial translocation-associated degradation
mPOS: Mitochondrial precursor overaccumulation stress
mtDNA: Mitochondrial DNA
MIA: IMS import and assembly machinery
MIM: Mitochondrial import complex
MTS: Mitochondrial targeting sequence
MPP: Mitochondrial protein peptidase
NLS: Nuclear localization signal
OMM: Outer mitochondrial membrane
ORF: Open reading frame
OXPHOS: Oxidative phosphorylation
PAM: Presequence translocase-associated motor
PD: Parkinson’s disease
Proteostasis: Protein homeostasis
ROS: Reactive oxygen species
RQC: Ribosome quality control
SAM: Sorting and assembly machinery
SMOC: Supramolecular organizing center
TCA: Tricarboxylic acid cycle
TIM: Translocase of the inner membrane
TOM: Translocase of the outer membrane
Ubl: Ubiquitin-like domain
TPR: Tetratricopeptide repeat
uORF: Upstream open reading frame
UPR^ER^: Unfolded protein response of the ER
UPRam: Unfolded protein response activated by the mistargeting of proteins
UPRmt: Mitochondrial unfolded protein response
UPS: Ubiquitin proteasome system
